# WRKY Transcription Factors in Response to Metal Stress in Plants: A Review

**DOI:** 10.3390/ijms252010952

**Published:** 2024-10-11

**Authors:** Yuanzhi Huang, Zhaofei Sun, Xiangui Zhou

**Affiliations:** College of Life Sciences and Oceanography, Shenzhen University, Shenzhen 518000, China; 11930164@mail.sustech.edu.cn (Y.H.); 2200251033@email.szu.edu.cn (Z.S.)

**Keywords:** aluminum, arsenate, cadmium, copper, WRKY TFs

## Abstract

Heavy metals in soil can inflict direct damage on plants growing within it, adversely affecting their growth height, root development, leaf area, and other physiological traits. To counteract the toxic impacts of heavy metals on plant growth and development, plants mitigate heavy metal stress through mechanisms such as metal chelation, vacuolar compartmentalization, regulation of transporters, and enhancement of antioxidant functions. WRKY transcription factors (TFs) play a crucial role in plant growth and development as well as in responses to both biotic and abiotic stresses; notably, heavy metal stress is classified as an abiotic stressor. An increasing number of studies have highlighted the significant role of WRKY proteins in regulating heavy metal stress across various levels. Upon the entry of heavy metal ions into plant root cells, the production of reactive oxygen species (ROS) is triggered, leading to the phosphorylation and activation of WRKY TFs through MAPK cascade signaling. Activated WRKY TFs then modulate various physiological processes by upregulating or downregulating the expression of downstream genes to confer heavy metal tolerance to plants. This review provides an overview of the research advancements regarding WRKY TFs in regulating heavy metal ion stress—including cadmium (Cd), arsenic (As), copper (Cu)—and aluminum (Al) toxicity.

## 1. Introduction

Soil plays a crucial role in ensuring food security [1]. As sessile organisms, plants are subjected to both biotic and abiotic stresses throughout their growth stages. Abiotic stressors encompass salt, drought, extreme temperatures, flooding, UV damage, and heavy metal stress [2]. Salt accumulation, drought conditions, extreme weather events, floods, and UV exposure can lead to significant reductions in crop yields or even total crop loss. In contrast to other abiotic stresses, heavy metal stress not only diminishes crop yields but also poses a severe threat to the quality of crops. These heavy metal ions accumulate in seeds and vegetative organs and can enter the human body directly or through the food chain, thereby posing substantial risks to human health [3]. The relevant heavy metal ions include cadmium (Cd), chromium (Cr), lead (Pb), copper (Cu), arsenic (As), and mercury (Hg) [4]. Heavy metal stress adversely affects seed germination and root development; damages antioxidant enzyme activity as well as cellular membrane integrity; induces chromosomal mutations; accelerates plant senescence; and may ultimately result in plant death. The impacts of heavy metal ions on plants primarily manifest as: (1) induction of reactive oxygen species (ROS) generation that alters antioxidant responses while stimulating oxidative stress; (2) direct interaction with specific functional protein groups such as sulfhydryl groups, carboxyl groups, and histidine residues, leading to conformational changes that render these proteins inactive; (3) displacement of essential cations from binding sites resulting in functional collapse; and (4) competition with certain nutrient ions for absorption at the root surface [5,6]. Plants overcome metal toxicity by limiting heavy metal ion absorption, vacuole compartmentalization, phytochelatin, hormone, metallothionein, enzyme, and non-enzymatic antioxidant synthesis [7].

WRKY TFs are characterized by the presence of a conserved WRKYGQK domain at their N-terminal region [8]. WRKY TFs represent one of the largest and most significant families of TFs in plants [9]. These factors are involved in the regulation of plant growth and development [10,11,12,13,14,15,16,17,18], disease resistance [19,20,21,22,23,24,25,26,27,28,29], and stress tolerance [30,31,32,33,34,35,36,37,38,39,40]. Numerous comprehensive review articles have highlighted the critical roles of WRKY TFs in modulating plant growth, disease resistance, and stress tolerance [41,42,43,44]. Despite a growing body of literature emphasizing the pivotal role of WRKY TFs in metal stress responses, there remains a lack of thorough reviews addressing their progress specifically concerning heavy metal stress. This article systematically elucidates the essential functions of WRKY TFs in mitigating heavy metal stress while also anticipating future research directions within this field.

## 2. Response of WRKY TFs to Cd Toxicity

The rapid advancement of modern industry and agriculture has resulted in a sustained increase in the production of metal cadmium, as anthropogenic activities have discharged substantial quantities of cadmium into aquatic environments, leading to severe cadmium pollution that poses significant risks to human health and ecological safety [45]. The presence of cadmium in soil primarily stems from airborne suspended soil particles and rock weathering. These particles originate from various natural phenomena, including forest fires, volcanic eruptions, and atmospheric dust [46]. In contrast, anthropogenic cadmium emissions predominantly arise from the application of phosphate fertilizers, tailings disposal, practices within the metal industry, mining operations, and fossil fuel combustion [47,48].

Due to variations in geographic location, as well as climatic conditions, the concentration of cadmium in soils across different countries and regions varies accordingly. Currently, the cadmium levels in soils worldwide have surpassed their original environmental background values. For instance, the average concentration of cadmium in European soils is 0.33 mg/kg [49], while in the United States, the average concentration is recorded at 0.265 mg/kg [50], and China exhibits a concentration of 0.19 mg/kg [51].

Cadmium, recognized as one of the most toxic heavy metal contaminants, not only adversely impacts plant growth and development but also poses significant risks to human health by entering the human body through the food chain [52,53,54]. Rice is one of the crops most significantly affected by cadmium contamination, with its germination, growth, and nutrient uptake all being influenced by cadmium [55]. To tackle the challenge of soil cadmium pollution, researchers have explored the mechanisms by which WRKY TFs mediate plant responses to cadmium stress across diverse species.

ROS, including superoxide radicals (O_2_^·−^), hydroxyl radicals (OH), and hydrogen peroxide (H_2_O_2_), function as signaling molecules within cells to mediate signal transduction [56]. Cadmium ions trigger the excessive production of ROS, resulting in oxidative damage to cells and ultimately detrimental effects on plant growth and development. Therefore, the timely removal of ROS by plant antioxidant enzymes such as superoxide dismutase (SOD), catalase (CAT), and peroxidase (POD) under cadmium stress is essential for enhancing plant tolerance to cadmium [57]. The Ascorbate-Glutathione (AsA-GSH) cycle effectively mitigates ROS levels. *TaWRKY74* alleviates cadmium toxicity by modulating the expression of AsA-GSH synthesis genes in wheat [58] (Table 1). Furthermore, *ZmWRKY4* confers resistance to cadmium detoxification in maize by upregulating the expression of *ascorbate peroxidase* (*APX*) and *superoxide dismutase 4* (*SOD4*), thereby enhancing antioxidant enzyme activity under cadmium stress [59]. Serving as a partner that interacts with transcription factors, members of the SIMILAR TO RCD-ONE (SRO) family also augment the capacity of plant antioxidant systems, providing resistance against drought and high-salinity stresses [60]. In potatoes, *StSRO5* and *StSRO6* confer tolerance to cadmium stress, with their expression potentially regulated by *StWRKY6* [61]. Consequently, *StWRKY6* may modulate potato cadmium tolerance through the regulation of the ROS system [61]. Indeed, heterologous expression of the *StWRKY6* gene in *Arabidopsis thaliana* enhances the activities of SOD, CAT, and POD, thereby mitigating ROS-induced damage under cadmium stress, a conclusion corroborated by reduced malondialdehyde (MDA) levels [62]. *ZmWRKY64* upregulates the expression of the ROS balance gene *ZmSRG7* in maize, conferring resistance to cadmium toxicity [63]. *BnaA10.WRKY75* regulates rapeseed’s tolerance to cadmium; its overexpression increases sensitivity to this metal stress, likely due to downregulation of *BnaC03.CAT2* expression and subsequent disruption of ROS homeostasis [64]. Under cadmium stress conditions, upregulation of *GmWRKY172* mitigates ROS damage in soybeans by regulating flavonoid and lignin biosynthesis while enhancing peroxidase activity [65]. In contrast to other WRKY TFs that exhibit negative regulatory effects, *CaWRKY41* plays a positive feedback role under cadmium stress in chili peppers. When subjected to such stress, hydrogen peroxide is produced and accumulates, promoting *CaWRKY41* expression. This factor subsequently stimulates genes involved in ROS production while inhibiting those encoding scavenging enzymes, thus maintaining elevated levels of ROS within chili peppers and exacerbating cadmium toxicity; however, it also enhances resistance against pathogens [66].

In addition to regulating the clearance of ROS, WRKY TFs can also detoxify cadmium by modulating processes such as chelation, transport, and sequestration of cadmium ions. Glutathione (GSH) and phytochelatin (PC) are capable of chelating cadmium ions, which are subsequently transported to vacuoles, thereby mitigating the toxicity of cadmium in *Arabidopsis* [67]. GSH is synthesized with the involvement of GSH1 and GSH2 and can be further converted into PC through the action of PCS1 and PCS2 [68,69,70]. *AtWRKY12* negatively regulates the synthesis of both GSH and PC. Like other WRKY TFs, *AtWRKY12* binds to the W-box region within the promoters of target genes; however, in this context, it inhibits their expression [67]. Notably, *AtWRKY12* does not simultaneously suppress all four genes—*GSH1*, *GSH2*, *PCS1*, and *PCS2*—but specifically binds to the promoter region of *GSH1* to inhibit its expression [67]. In contrast to *AtWRKY12*, *AtWRKY45* exhibits enhanced expression under cadmium stress and is capable of binding to W-box elements in the promoters of *PCS1* and *PCS2* genes to promote their expression, thus facilitating PC synthesis [71]. *PyWRKY48* also confers cadmium tolerance in poplar primarily by enhancing activities related to GSH and PCs [72]. The process by which cells transport cadmium ions into vacuoles necessitates participation from specific transporters located on vacuolar membranes [73]. Heavy metal ATPase 3 (HMA3) and V-type ATPase c subunit ThVHAc are localized transporters that facilitate cadmium uptake into vacuoles [74]. *ScWRKY35* positively regulates *ScHMA3* expression, thereby sequestering cadmium within *Salvia castanea* Diels roots; additionally, *ThWRKY7* may function as a transcriptional activator for *ThVHAc1* under conditions of cadmium stress by promoting its expression in *Tamarix hispida* [75,76]. Cation/H^+^ exchanger (CAX), another transporter found on vacuolar membranes, is potentially involved in vacuolar transport mechanisms for cadmium regulation. *StWRKY6* may regulate *StCAX1* and *StCAX4* expressions during exposure to cadmium stress in potatoes [77].

Additionally, cells can mitigate cadmium toxicity by preventing the entry of cadmium ions. CDT1 is a family of cysteine-rich peptides located on the cell membrane that can chelate cadmium ions, thereby inhibiting their further uptake into cells [78]. Under conditions of cadmium stress, the expression of the *GmWRKY142* gene is upregulated and binds to the W-box regions within the promoters of *GmCDT1-1* and *GmCDT1-2*, activating their expression [79]. Inter-organ transport among different organs or tissues also contributes to enhanced plant tolerance to cadmium. For instance, soybean *GmWRKY172* reduces the transport of cadmium ions from roots to shoots and seeds, thereby improving overall cadmium tolerance [65]. Meanwhile, researchers have identified a nuclear-localized gene, *ZmWRKY64*, whose expression in maize leaves and roots increases under cadmium stress. Knockout experiments involving this gene result in heightened accumulation of cadmium in both leaves and roots while exhibiting a hypersensitive phenotype towards this metal. When present, *ZmWRKY64* upregulates a series of genes involved in cadmium translocation [63].

**Table 1:** WRKY and downstream genes in response to cadmium stress.

Species	WRKY Genes	Downstream Genes	Regulatory Types	Reference
*A. thaliana*	*AtWRKY12*	*AtGSH1*	Negative	[67]
	*AtWRKY13*	*AtPDR8*	Positive	[80]
	*AtWRKY33*	*AtATL31*	Positive	[81]
	*AtWRKY45*	*AtPCS1* and *AtPCS2*	Positive	[71]
*Zea mays*	*ZmWRKY64*	*ZmSRG7*, *ZmABCC4*, *ZmHMA3*, *ZmNRAMP5*, *ZmPIN2*, *ZmABCG51*, *ZmABCB13*, *ZmABCB32*, and *ZmABCB10*	Positive	[63]
	*ZmWRKY4*	*ZmcAPX* and *ZmSOD4*	Positive	[59]
*Glycine max*	*GmWRKY142*	*GmCDT1-1* and *GmCDT1-2*	Positive	[79]
	*GmWRKY172*	*-*	-	[65]
*Solanum tuberosum*	*StWRKY6*	*StSRO5*, *StSRO6*, *StCAX1*, and *StCAX4*	Positive	[61,77]
*Populus yunnanensis*	*PyWRKY48*	*PaGRP*, *PaPER*, and *PaPHOS*	Positive	[72]
	*PyWRKY71*	*-*	-	[82]
	*PyWRKY75*	-	-	[83]
*Triticum aestivum*	*TaWRKY74*	*TaGSH*, *TaGPX*, *TaGR*, *TaDHAR*, *TaMDHAR*, and *TaAPX*	Positive	[58]
		*TaNramp1*, *TaNramp5*, *TaHMA2*, *TaHMA3*, *TaLCT1*, and *TaIRT1*	Negative	
	*TaWRKY70*	*TaCAT5*	Positive	[84]
*Capsicum annuum*	*CaWRKY41*	*CaCAT1*, *CaSOD1*, *CaCSD2*, *CaAPX1*, and *CaAPX2*	Negative	[66]
*T. hispida*	*ThWRKY7*	*ThVHAc1*	Positive	[76]
*Brassica napus*	*Bna10.WRKY75*	*BnaC03.CAT2*	Positive	[64]
		*BnaC03.HMA4c*	Negative	
*S. castanea*	*ScWRKY35*	*ScHMA3*	Positive	[75]
		*ScNRAMP1*	Negative	

The regulation of cellular physiological processes, such as the uptake and efflux of cadmium ions by WRKY TFs, serves as a mechanism to mitigate cadmium toxicity. *AtWRKY13* can bind to the promoter region of the ABC transporter *PLEIOTROPIC DRUG RESISTANCE8* (*PDR8*), thereby activating its transcription. Once expressed, PDR8 functions as a pump to expel cadmium ions from *Arabidopsis*, consequently reducing cadmium accumulation in this species [80]. The IRON-REGULATED TRANSPORTER 1 (IRT1) is a plasma membrane-localized cadmium transporter that facilitates the uptake of cadmium into cells. Under conditions of cadmium stress, *AtWRKY33* induces upregulation of *Arabidopsis Tóxicos en Levadura 31* (*ATL31*) expression; *ATL31* subsequently promotes ubiquitin-conjugated degradation of IRT1 through direct interaction, thereby decreasing cadmium ion absorption [81]. Furthermore, heterologous expression of *ZmWRKY64* in *Arabidopsis* downregulates *AtIRT1* and *AtZIP1* gene expressions to inhibit cadmium absorption while suppressing *AtHMA2* expression to block transport pathways, ultimately achieving reduced cadmium accumulation [63]. In poplar, researchers have cloned *PyWRKY71* and *PyWRKY75* genes that enhance both absorption and accumulation of environmental cadmium, thus playing a purifying role against pollution. However, these genes also increase activities of antioxidant enzymes such as superoxide dismutase (SOD), peroxidase (POD), and catalase (CAT), thereby enhancing poplar’s tolerance to cadmium toxicity [82,83]. *TaWRKY70* can directly bind to the promoter of the cationic amino acid transporter (CAT) *TaCAT5*, initiating its expression and conferring cadmium tolerance to wheat. Heterologous expression of *TaWRKY70* in *Arabidopsis* reduces cadmium ion uptake while enhancing antioxidant enzyme activity [84]. In the roots of *S. castanea* Diels, *ScWRKY35* reduces cadmium absorption by downregulating the expression of the cadmium uptake gene *Natural Resistance-Associated Macrophage Protein 1* (*NRAMP1*) [75]. Treatment with polyaspartic acid (PASP) under conditions of cadmium stress in *Solanum nigrum* L. enhances both accumulation and transport of cadmium [85], while transcriptomic analysis indicates that WRKY TFs’ expressions are downregulated following PASP treatment, suggesting their crucial role in *S. nigrum*’s accumulation and translocation of cadmium [86].

Additionally, studies in various species, including tobacco, *Populus simonii* × *Populus nigra*, castor bean, tomatoes, and muskmelon, have demonstrated that WRKY TFs are involved in plant responses to cadmium stress, and indicated that the regulation of cadmium tolerance in plants by WRKY TFs is a widespread phenomenon, although the specific mechanisms remain to be further elucidated [87,88,89,90,91]. Furthermore, the regulation of WRKY TF genes occurs via m6A methylation in barley under conditions of cadmium stress, providing new insights into the regulatory mechanisms governing their expression [92].

## 3. Response of WRKY TFs to As Toxicity

Arsenic, as a natural element in the earth’s crust, has sources of contamination including contaminated groundwater, metal smelting, fossil fuel combustion, pesticide, and fungicide production [93,94]. China is a country with extensive arsenic pollution, mainly because the rapid development of Chinese industry has led to the release of large amounts of arsenic into the soil over the past few decades, and rice, a staple food for Chinese, is particularly affected by arsenic pollution [95]. Arsenic enters plants through root absorption and can have detrimental effects on plant germination, plant height, and root length [96].

In the environment, arsenate [As(V)] is a prevalent form of arsenic and a chemical analog of phosphate (Pi), allowing it to be transported into *Arabidopsis* via the Pi transporter PHOSPHATE TRANSPORTER1;1 (PHT1;1) [97]. *AtWRKY6* is an As(V)-responsive gene that regulates As(V) uptake [98]. Under conditions of As(V) stress, *AtWRKY6* inhibits the expression of *PHT1;1*, leading to its delocalization from the plasma membrane and consequently reducing As(V) uptake by *Arabidopsis*. Once As(V) stress is alleviated, PHT1;1 resumes normal expression and relocalizes to the plasma membrane to fulfill its role in Pi transport [98] (Table 2). Additionally, transcriptional activation of transposons induced by As(V) is restricted by *AtWRKY6* [98]. The activation of transposons can result in chromosomal rearrangements, gene deletions or insertions, and alterations in gene expression, potentially having detrimental effects on plants [99,100]. Typically, stress-induced transposon activation is primarily suppressed through epigenetic mechanisms [100,101,102]. Acting as a transcriptional repressor, *AtWRKY6* promotes transposon silencing in response to As(V) stress, representing a novel survival strategy employed by plants under such conditions [98].

In rice, *OsWRKY28* also participates in the transport of As(V), with its expression rapidly induced by this compound. In *OsWRKY28* mutants, concentrations of As(V) in shoots are significantly lower than those found in roots; however, no difference exists between mutant and wild-type concentrations within root tissues. Furthermore, the mutation of *OsWRKY28* does not affect the expression levels of Pi transporters, suggesting that *OsWRKY28* may facilitate upward transport of As(V) within rice without influencing its absorption capabilities [103]. Interestingly, arsenic stress triggers the MAPK signaling cascade and activates *OsWRKY76* through phosphorylation [104,105]. The activated *OsWRKY76* subsequently binds to the promoters of iron transporters *OsIRT1* and *Oryza sativa Yellow Stripe-Like 2* (*OsYSL2*), suppressing their expression and resulting in reduced iron absorption as well as disruption of iron homeostasis in rice. However, supplementation with exogenous iron under arsenic stress downregulates *OsWRKY76* expression, which promotes the expression of *OsIRT1* and *OsYSL2*, thereby restoring iron absorption [105].

**Table 2:** WRKY and downstream genes in response to arsenate stress.

Species	WRKY Genes	Downstream Genes	Regulatory Types	Reference
*A. thaliana*	*AtWRKY6*	*AtPHT1;1*	Negative	[98]
*Oryza sativa*	*OsWRKY28*	-	-	[103]
	*OsWRKY76*	*OsIRT1* and *OsYSL2*	Negative	[105]
	*OsWRKY71*	-	-	[106]
*G. max*	*GmWRKY6 GmWRKY46 GmWRKY56 GmWRKY106*	-	-	[107]

A recent study has demonstrated that exogenous Fe supplementation under arsenic stress can also enhance root system architecture (RSA), primarily through regulation by *OsWRKY71*. Arsenic induces alterations in RSA in rice, thereby limiting growth and yield. Following Fe supplementation, the expression of *OsWRKY71* is upregulated; additionally, the interaction between *OsWRKY71* and the DELLA protein SLR1 from the gibberellin pathway suggests that *OsWRKY71* may facilitate improvements in RSA by Fe under arsenic stress via GA signaling pathways [106].

In soybeans, researchers treated the plants with zinc oxide nanoparticles (ZnONP) and/or selenium nanoparticles (SeNP), resulting in increased levels of heavy metal chelators such as GSH and phytochelatin (PC), along with reduced ROS content. Additionally, arsenic stress was found to upregulate the expression of *GmWRKY6*, *GmWRKY46*, *GmWRKY56*, and *GmWRKY106*; ZnONP and SeNP also induce the expression of these genes. Therefore, these WRKY TFs may confer tolerance to soybeans under arsenic stress by enhancing the expression of chelators and increasing the activity of ROS-scavenging enzymes [107].

## 4. Response of WRKY TFs to Al Toxicity

Aluminum is the most abundant metal element on Earth, existing in large quantities in rocks and entering the environment through natural weathering processes as well as human activities such as coal combustion, mining, and air emissions [108]. The world’s acidic soils are predominantly found in the hot and humid tropical regions of the Southern Hemisphere and the cold and humid temperate zones of the Northern Hemisphere [109]. More than 50% of the world’s potentially arable land consists of acidic soils, and when soil pH is equal to or below 5, aluminum (Al) exists in its toxic form as Al^3+^. Even at low concentrations, Al^3+^ rapidly inhibits root elongation [110]. Al also exerts toxicity on physiological processes in plants, including photosynthetic efficiency, metabolism, and the absorption of mineral nutrition [111].

Plants employ two primary strategies to cope with Al toxicity: external exclusion and internal detoxification. In external exclusion, plants secrete organic acid anions such as malate, oxalate, and citrate at the root tips; these anionic acids chelate Al outside the cells, preventing its entry and thus averting toxicity [112,113]. The aluminum (Al)-activated malate transporter 1 (*ALMT1*), an apple acid transporter, facilitates the secretion of malate produced by plants outside the cell to chelate aluminum. This process also confers Al tolerance to barley and *Arabidopsis* [114]. In *Arabidopsis*, *AtWRKY46* acts as a negative regulator of *ALMT1* by binding to the W-box region of the *ALMT1* gene and inhibiting its expression; this suppression reduces malate secretion. However, under Al stress conditions, *AtWRKY46* expression is inhibited, allowing normal expression of *ALMT1* and subsequent secretion of malate that enables *Arabidopsis* to resist Al toxicity [114] (Table 3). In tomatoes, *SlWRKY42* negatively regulates *SlALMT9* expression. Mutants lacking functional *SlALMT9* exhibit high malate content due to mutations in the W-box region of the *SlALMT9* promoter that prevent binding by *SlWRKY42*. Additionally, under Al stress, the abundance of *SlALMT9* localized to vacuolar membranes increases, enhancing both malate transport and resistance against aluminum; this indicates that *SlWRKY42* expression is suppressed under such conditions [115]. Similarly, in both *Arabidopsis* and tomato, jasmonic acid (JA) enhances inhibition on tomato root growth caused by aluminum while suggesting that *SlALMT3*—a key protein involved in crosstalk between regulatory mechanisms for aluminum and JA—may be regulated by SIWRKYs acting as upstream regulatory proteins within this interaction mechanism [116].

In rice, *OsFRDL4*, a citrate transporter, is regulated by the *ALUMINUM* (*Al*) *RESISTANCE TRANSCRIPTION FACTOR 1* (*ART1*) as well as *OsWRKY22*. Under Al stress, mutants of *OsWRKY22* exhibit increased sensitivity to Al and reduced Al-induced citrate secretion, indicating that *OsWRKY22* positively regulates the expression of *OsFRDL4* and citrate secretion under such conditions; its upregulation enhances rice’s tolerance to Al [117]. In sorghum, Sorghum bicolor multidrug and toxic compound extrusion (*SbMATE*) also functions as a citrate transporter, conferring Al tolerance to the plant. Chromatin immunoprecipitation (ChIP) analyses have revealed that *SbMATE* is regulated by *SbWRKY* TFs; further haplotype analysis suggests that these WRKY TFs can influence *SbMATE* expression to regulate Al tolerance [118]. However, specific WRKY TFs regulating *SbMATE* require further investigation.

**Table 3:** WRKY and downstream genes in response to aluminum stress.

Species	WRKY Genes	Downstream Genes	Regulatory Types	Reference
*A. thaliana*	*AtWRKY46*	*AtALMT1*	Negative	[114]
	*AtWRKY47*	*AtELP* and *AtXTH17*	Positive	[119]
*Solanum lycopersicum*	*SlWRKY42*	*SlALMT9*	Negative	[115]
*Oryza sativa*	*OsWRKY22*	*OsFRDL4*	Positive	[117]
*Sorghum bicolor*	*SbWRKY22*	*SbMATE*, *SbGlu1*, *SbSTAR1*, *SbSTAR2a*, and *SbSTAR2b*	Positive	[120]
	*SbWRKY65*	*SbWRKY22*	Positive	[120]
*G. max*	*GmWRKY21*	*GmCOR47*, *GmDREB2A*, *GmMYB84*, *GmKIN1*, *GmGST1*, and *GmLEA*	Positive	[121]
	*GmWRKY81*	genes related to Al^3+^ transport, organic acid secretion, and antioxidant reactions	-	[122]

In addition to secreting organic acid anions to cope with aluminum (Al) stress, plants can also modify the properties of their cell walls to enhance Al tolerance. *EXTENSIN-LIKE PROTEIN* (*ELP*) and *XYLOGLUCAN ENDOTRANSGLUCOSYLASE-HYDROLASES17* (*XTH17*) genes are involved in modifying the cell wall, while *WRKY47* regulates the expression of these two genes to modulate plant tolerance to Al stress. The absence of *AtWRKY47* results in a decrease in hemicellulose I content, thereby reducing the cell wall’s capacity to bind Al [119]. When heterologous expressed in *Arabidopsis*, sweet sorghum *SbWRKY22*, and *SbWRKY65* enhance aluminum tolerance, which is associated with callose deposition in the roots [120].

In soybean, *GmWRKY21* responds to aluminum stress and is induced for expression, potentially promoting Al stress tolerance by regulating genes responsive to both aluminum and abiotic stresses. Heterologous expression of *GmWRKY21* in *Arabidopsis* facilitates root growth under Al stress, increases proline content, and reduces MDA levels [121]. Additionally, *GmWRKY81* can also be induced by Al stress; its overexpression enhances soybeans’ tolerance to Al, likely through the regulation of aluminum transport, organic acid secretion, and antioxidant enzyme genes [122]. In tea plants and millet, studies have shown that WRKY proteins also participate in the regulation of Al tolerance [123,124], indicating the universality of WRKY-mediated Al tolerance across species.

## 5. Response of WRKY TFs to Cu Toxicity

Copper is essential for plant growth and development; however, excessive copper in the soil adversely affects plant growth, development, and yield while accumulating in plants and posing health risks to humans through the food chain [125]. The overaccumulation of copper in soil primarily results from the use of fungicides, copper-containing fertilizers, and wastewater irrigation [126]. Rice fields may be more susceptible to copper contamination because organic matter and other components in the upper soil of rice fields are more likely to attach copper, making it less likely to migrate [127].

To mitigate the detrimental effects of copper stress on plants, two strategies—copper efflux and copper sequestration—are employed by plants to remove excess copper from critical tissues [128]. In copper efflux, copper ions are transported from the cytoplasm of root epidermal cells to the cell wall for storage outside the cell, thereby maintaining normal intracellular copper levels. Copper sequestration involves chelating copper ions with specific proteins that transport them out of the cell and subsequently up through the root for storage in stem tissues [129].

HMA5 is a Cu-specific P1B-type ATPase localized at the plasma membrane of root epidermal cells responsible for transporting copper ions out of these cells [130,131]. In apple trees, *MdWRKY11* responds to copper stress and is induced for expression. Overexpression of *MdWRKY11* confers copper stress tolerance to apple trees, primarily because it activates the expression of *MdHMA5*, facilitating the transport of excessive copper ions out of the cell and thereby alleviating their adverse effects on normal physiological processes [132] (Table 4).

Beyond merely removing excess copper, plants also require detoxification mechanisms against copper toxicity, which involve both enzymatic and nonenzymatic forms [133]. GSH plays a crucial role as a metal chelator in the nonenzymatic detoxification of copper [134]. In wheat, *TaWRKY74* regulates the expression of the key GSH synthesis enzyme *TaGST1*. Silencing *TaWRKY74* results in decreased GSH content in wheat roots, accompanied by increased levels of MDA, H_2_O_2_, and copper. Therefore, *TaWRKY74* significantly contributes to copper tolerance in wheat by modulating GSH synthesis [135].

In rice, *OsWRKY37* is involved in regulating flowering time and grain fertility under conditions of copper deficiency by activating the expression of *OsCOPT6* and *OsYSL16*. Although its specific role under copper stress remains unclear, *OsWRKY37* is implicated in processes such as uptake, translocation from roots to shoots, and distribution within plants; thus, suggesting that it may respond to copper stress [136]. Additionally, *OsWRKY11* can be induced by excessive levels of copper in rice, indicating its involvement in coping with this form of stress [137].

**Table 4:** WRKY and downstream genes in response to copper, iron, or mercury stress.

Species	WRKY Genes	Downstream Genes	Regulatory Types	Metal Stress	Reference
*Malus domestica*	*MdWRKY11*	*MdHMA5*	Positive	Copper	[132]
*T. aestivum*	*TaWRKY74*	*TaGST1*	Positive		[135]
*O. sativa*	*OsWRKY11*	-	-		[137]
*O. sativa*	*OsWRKY55-like* *OsWRKY46* *OsWRKY64* *OsWRKY113*	-	-	Iron	[138]
	*OsWRKY80*	-	-		[139]
*A. thaliana*	*AtWRKY46*	*AtVITL1*	Negative		[140]
*T. aestivum*	*TaWRKY19*	-	-	Mercury	[141]

**Figure 1:**
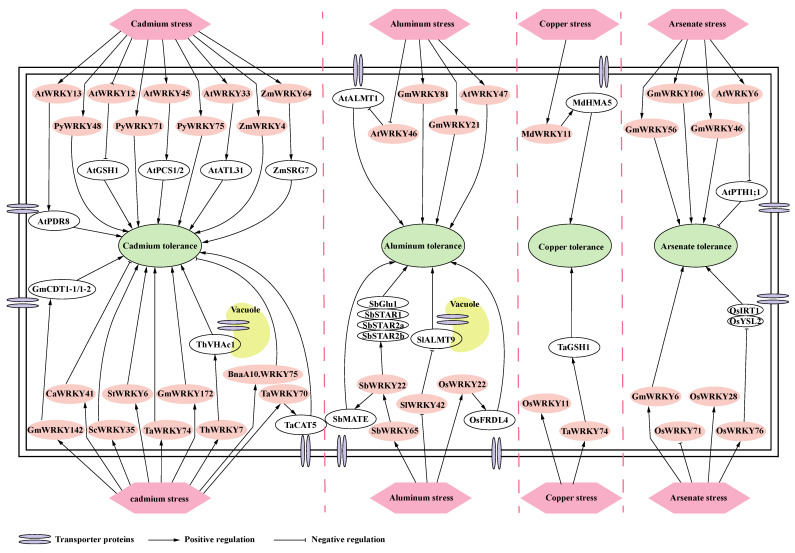
WRKY genes are involved in regulatory pathways of plant tolerance against metal stress. After responding to cadmium, aluminum, copper, and arsenate stress, WRKY TFs directly bind to the promoters of downstream genes to activate or inhibit their expression, thereby conferring metal stress tolerance or sensitivity to plants. Downstream genes not directly regulated by WRKY transcription factors are not marked, and WRKY genes without function or with unclear function in plant tolerance are not indicated with regulatory relationships in the diagram.

In summary, we have delineated the regulatory network of key WRKY TFs in response to cadmium, aluminum, copper, and arsenic ion toxicity (Figure 1).

## 6. Response of WRKY TFs to Other Metal Toxicity

Research on the regulatory relationships between WRKY TFs and cadmium, arsenic, aluminum, and copper stress are relatively abundant; however, studies examining their regulatory interactions with other metal stresses are comparatively scarce.

Mercury affects the oxidative metabolism of wheat by increasing hydrogen peroxide, MDA, and proline levels while decreasing SOD and CAT activities. The downregulation of *TaWRKY19* in response to mercury stress suggests its involvement in regulating the ROS system [141].

In rice, *OsWRKY55-like*, *OsWRKY46*, *OsWRKY64*, *OsWRKY113*, and *OsWRKY80* respond to iron stress [138,139], indicating that WRKY TFs play a crucial role in iron homeostasis. Besides responding to excessive iron stress, WRKY TFs also react to iron deficiency. In *Arabidopsis*, *AtWRWY46* is involved in responses to iron deficiency and regulates transport from roots to shoots primarily through negative regulation of *VACUOLAR IRON TRANSPORTER1-LIKE1* (*VITL1*). The absence of *AtWRKY46* coupled with upregulation of *VITL1* expression leads to increased transport of iron into vacuoles while decreasing transport to shoots—resulting in leaf yellowing under conditions of iron deficiency [140].

High concentrations of Cr(VI) are toxic to plants as they induce ROS production in rice and upregulate WRKY TFs; however, the specific roles of these factors under Cr(VI) stress require further investigation [142].

Manganese toxicity—a limiting factor for crop production in acidic soils—inhibits plant iron accumulation and causes oxidative damage; transcriptome analysis indicates that WRKY TFs are regulated by manganese stress but necessitates further elucidation regarding their specific roles under such conditions [143,144].

## 7. Conclusions and Future Prospects

**Figure 2:**
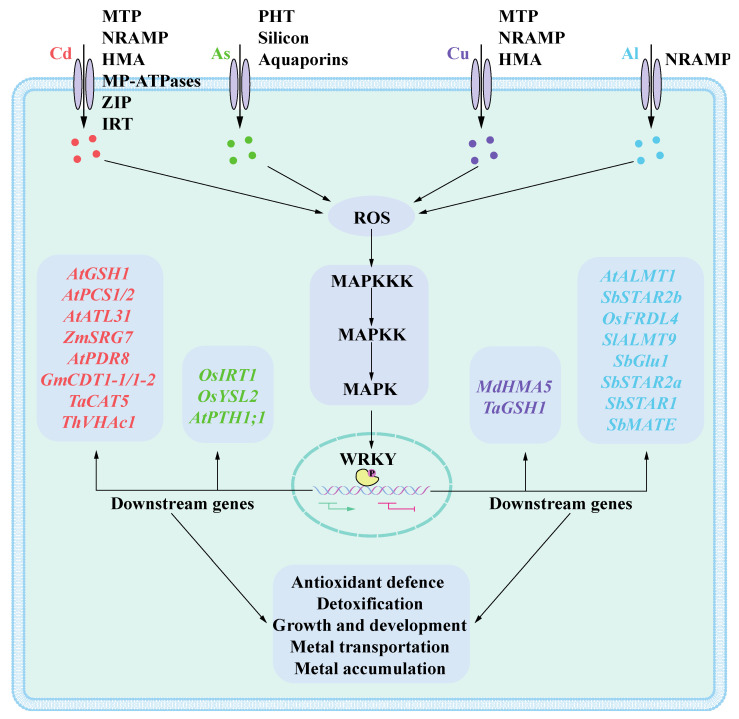
Mechanism of metal stress tolerance regulated by WRKY TFs in plants. When cadmium (Cd), aluminum (Al), copper (Cu), and arsenate (As) ions are transported into cells via membrane transporters, they induce the production of ROS, which further triggers the MAPK cascade signaling pathway, ultimately phosphorylating WRKY TFs and activating them. The WRKY TFs regulates various physiological processes through activating or repressing the expression of downstream genes by binding to their promoters, thereby conferring metal stress tolerance in plants. Abbreviations of membrane transporters are as follows: ZIP (zinc–regulated transporter), IRT (iron–regulated transporter), MP–ATPases (metal–pumping ATPases), PHT (phosphate transporter), HMA (heavy metal ATPase), MTP (metal tolerance protein), NRAMP (natural resistance associated macrophage protein).

Nitrogen (N), phosphorus (P), and potassium (K) are essential macronutrients for plant growth and development. Certain micronutrients, including zinc (Zn), iron (Fe), manganese (Mn), molybdenum (Mo), and copper (Cu), play critical roles in various physiological processes within plants, serving as vital components of metalloproteins and ion-dependent enzymes [145]. However, when the concentrations of these metals exceed the optimal levels necessary for plant metabolism, they can become toxic. Conversely, other heavy metals such as cadmium (Cd), mercury (Hg), and lead (Pb) are non-essential to plant metabolism; even at minimal concentrations, they can have detrimental effects [146]. The accumulation of heavy metals in soil is exacerbated by chemical waste from industrial activities and agricultural runoff—including fertilizers, herbicides, and pesticides—leading to elevated metal concentrations. Due to their non-degradable nature, heavy metals pose long-term risks to ecosystem health [147]. As one of the largest families of TFs, WRKY TFs play a crucial role in normal physiological processes in plants. Research on WRKY TFs has expanded significantly, revealing that they not only contribute to plant growth and development but also exhibit complex regulatory mechanisms while being involved in networks related to both biotic and abiotic stressors. Currently, there are still relatively few reports on how WRKY proteins mitigate heavy metal stress. We have summarized the regulatory model of WRKY TFs in responding to cadmium, aluminum, copper, and arsenic ion toxicity (Figure 2). Furthermore, the mechanisms underlying signal transmission between metal stress-induced perception and WRKY response remain inadequately characterized. The functions of numerous WRKY TFs have been validated in model organisms, providing a solid theoretical foundation for studies on other plant species. However, investigations into the roles of WRKY proteins in crops remain limited. Given that crops frequently encounter various stresses, further research is essential to explore WRKY TFs across a broader range of crop species. In light of severe environmental changes, utilizing WRKY TFs to identify stress-resistant plant varieties and enhance their resilience will significantly benefit agricultural yield and quality.
